# Rare case of mantle cell lymphoma of the lacrimal sac

**DOI:** 10.5935/0004-2749.2023-0205

**Published:** 2024-07-09

**Authors:** Mehmet Can Özen, Özlen Rodop Özgür, Seyhan Kocabaş

**Affiliations:** 1 Department of Ophthalmology, Şişli Hamidiye Etfal Education and Research Hospital, İstanbul, Turkey; 2 Department of Ophthalmology, Bayındır İçerenköy Hospital, İstanbul, Turkey; 3 Department of Ophthalmology, Dünyagöz Adana Eye Hospital, Adana, Turkey

**Keywords:** Lymphoma, mantle cell, Nasolacrimal duct, Neoplasms, Neoplasm staging, Dacryocystorhinostomy, Diagnosis, differential

## Abstract

Mantle cell lymphoma of the ocular and periorbital regions is extremely rare but should be considered in the differential diagnosis of lesions affecting the periorbital tissues. In this study, we present a rare case of mantle cell lymphoma of the lacrimal sac in a 65-year-old male presenting with a mass in the lacrimal sac region and epiphora. After clinical examinations and imaging studies, the mucocele was misdiagnosed. Considering the unexpected findings during external dacryocystorhinostomy, a frozen biopsy was performed, which confirmed the diagnosis of lymphoma.

## INTRODUCTION

Lacrimal system tumors are rare, and most have an epithelial origin (90%). Lacrimal sac lymphoma is a nonepithelial tumor that accounts for 6% of all lacrimal sac tumors. Most reported cases involve secondary involvement of systemic lymphoproliferative disease^(1)^.

To date, only three studies on mantle cell lymphoma (MCL) in the lacrimal sac have been published^(2,3,4)^. In this study, we aimed to present a case that was initially misdiagnosed as mucocele but was diagnosed as lymphoma via frozen biopsy that was performed during external dacryocystorhinostomy (DCR) due to its abnormal structure.

## CASE REPORT

**Figure 1:**
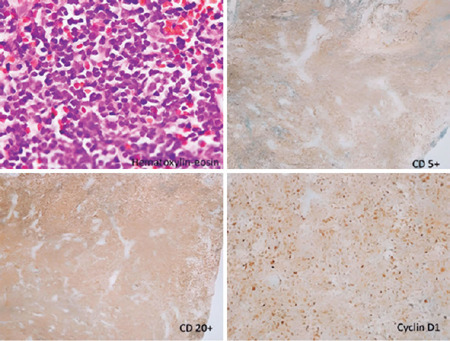
Orbital CT imaging shows a well-defined hypodense soft tissue mass in the right medial orbit (18 × 11 mm) not extending to the surrounding tissues.

A 65-year-old male complained of epiphora and swelling in the right medial canthal region for approximately 3 months. After reviewing the patient’s medical records, a mass was recorded three months ago, but the lacrimal irrigation was patent. The patient did not undergo further examination. During the examination, a semimotile, nontender mass, measuring approximately 10 × 15 mm, was detected. Diagnostic lacrimal irrigation tests revealed a hard stop an obstruction. The patient’s best-corrected visual acuity was 20/20 in both eyes and the anterior and posterior segments were normal based on biomicroscopic examination. Orbital computed tomography (CT) revealed a well-circumscribed hypodense soft tissue mass in the right medial orbit (18 × 11 mm) that did not spread to the surrounding tissues (Figure 1).

An external DCR was planned. During the surgery, a polypoidal lesion of approximately 2 × 3 mm was observed in the palpebral conjunctiva, and an excisional biopsy was performed on the mass. The lacrimal sac was filled with pink, lobulated tissue. Intraoperative frozen section revealed lymphoid cell proliferation with small round nuclei, absence of nucleoli, and scant cytoplasm with scattered and nodular patterns, which is highly suggestive of lymphoma. To confirm the histopathological results, the lacrimal sac and nasolacrimal duct were completely excised, bicanalicular silicone tube intubation was performed, and the nasal mucosal flap was sutured to subcutaneous tissue.

**Figure 2:**
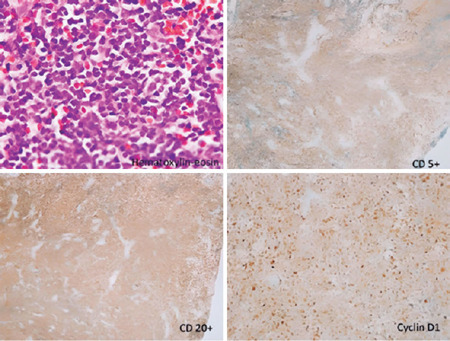
Hematoxylin–eosin staining shows infiltration by small me­dium-sized lymphocytes with small, round nuclei and scant cytoplasm, mitotic state, and diffuse and partially nodular growth pattern (x200). Immunohistochemistry is positive for CD5, CD20, and cyclin D1.

**Figure 3:**
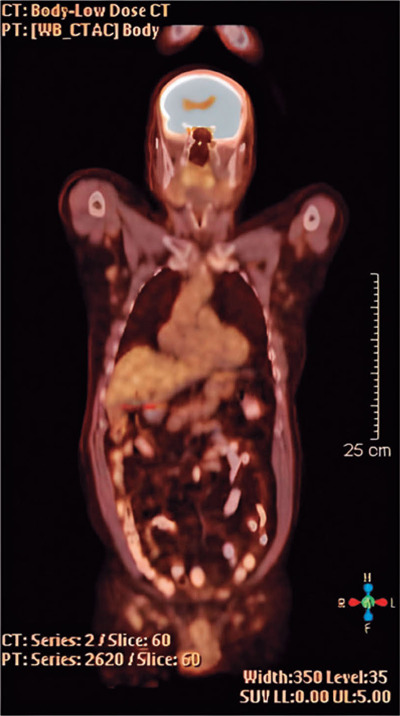
PET scan indicating multiple lymphadenopathies showing increased fludeoxyglucose uptake in the mediastinum (11 mm), left axillary fossa (14 mm), and infra- and supra-diaphragmatic paraaortic areas (19 mm).

Histopathological and immunohistochemical findings revealed classical MCL (Figure 2). The patient was referred to the oncology department. Additional tests such as those for liver function, erythrocyte sedimentation rate, blood urea nitrogen level, creatinine level, β2 microglobulin level, lactate dehydrogenase level, leukocyte count, whole-body positron emission tomography (PET) (Figure 3), and bone marrow biopsy were performed.

The PET scan revealed multiple lymphadenopathies in the mediastinum, axillary fossa, and infra- and supra-diaphragmatic paraaortic areas. Bone marrow examination revealed involvement of the bone marrow. The patient was classified as having T_4_N_3_M_1c_ disease according to the TNM staging system for ocular adnexal lymphoma (Table 1).

**Table 1:** TNM staging of ocular adnexal lymphoma (eighth edition)

Category	Definition
Primary tumor (T)	TX	T0	T1	T2	T3	T4	*Lymphoma extend not specified*	*No evidence of lymphoma*	*Lymphoma involving the conjunctiva alone without eyelid or orbital involvement*	*Lymphoma with orbital involvement with or without conjunctival involvement*	*Lymphoma with preseptal eyelid involvement with or without orbital involvement and with or without conjunctival involvement*	*Orbital adnexal lymphoma and extra-orbital lymphoma extending beyond the orbit to adjacent structures, such as the bone, maxillofacial sinuses, and brain*
Regional lymph nodes (N)	NX	N0	N1	N1a	N1b	N2	N3	Involvement of lymph nodes not assessed	No evidence of lymph node involvement	Involvement of lymph node region or regions draining the ocular adnexal structures and superior to the mediastinum (periauricular, parotid, submandibular and cervical nodes)	Involvement of the single lymph node region superior to the mediastinumMetastasis in a single ipsilateral regional lymph node based on lymph node biopsy	Metastasis in a single ipsilateral regional lymph node, >3 cm in greatest dimension, or in bilateral or contralateral lymph nodes	Diffuse or disseminated involvement of peripheral and central lymph node regions
Distant metastasis (M) M0	No evidence of involvement of other extranodal sites
M1a	M1b	M1c	Noncontiguous involvement of tissues or organs external to the ocular adnexa (e.g., parotid glands, submandibular gland, lung, liver, spleen, kidney, breast)	Lymphomatous involvement of the bone marrow	M1a and M1b involvement

Source: AJCC: American Joint Committee on Cancer.

TNM Staging of Ocular Adnexal Lymphoma. 8^th^ ed. AJCC.

The patient was treated with six courses of rituximab, cyclophosphamide, doxorubicin, vincristine, and prednisone (R-CHOP) by the oncology department. Regression of systemic lesions after six cycles of chemotherapy was revealed in a PET re-evaluation. Written informed consent for publication was obtained from the patient.

## DISCUSSION

Tumors arising in the lacrimal sac are extremely rare and usually occur during the fifth decade of life. They constitute 2.6% of all lacrimal system obstructions^(4,5)^.

The most common presentation of lacrimal sac lymphomas is similar to that of nontumor cases of lacrimal system obstruction, which is epiphora and painless or painful medial canthal swelling^(1,5)^.In 1956, Jones described the clinical symptoms of lacrimal tumors as epiphora, dacryocystitis, masses, and bleeding^(6)^.

Up to 40% of lacrimal tumors are not suspected via medical examination and are diagnosed during DCR.^(2)^ In our case, the diagnosis was made while performing DCR.

MCL represents only 3%–10% of all no Hodgkin’s lymphomas. It has been reported very rarely in the periocular region. However, periocular involvement is more common in the orbit and eyelids. It is usually obser­ved in men over 60 years of age and is an aggressive tumor. One- to two-thirds of the patients had a history of lymphoma, and for the remainder, the diagnosis was made when the disease was already widespread^(2)^.

In conclusion, malignancy should be considered in the differential diagnosis of patients presenting with a mass in the lacrimal sac region and epiphora. Moreover, rapid imaging and clinical and histopathological tests should be conducted to confirm the diagnosis, and patients should be referred to the oncology department for systemic evaluation and treatment. MCL with secondary involvement of the lacrimal sac is rare and usually has poor prognosis. Furthermore, early diagnosis and patient referral for systemic evaluation and treatment can prolong the survival rates.
